# Management of patients with multiple myeloma beyond the clinical-trial setting: understanding the balance between efficacy, safety and tolerability, and quality of life

**DOI:** 10.1038/s41408-021-00432-4

**Published:** 2021-02-18

**Authors:** Evangelos Terpos, Joseph Mikhael, Roman Hajek, Ajai Chari, Sonja Zweegman, Hans C. Lee, María-Victoria Mateos, Alessandra Larocca, Karthik Ramasamy, Martin Kaiser, Gordon Cook, Katja C. Weisel, Caitlin L. Costello, Jennifer Elliott, Antonio Palumbo, Saad Z. Usmani

**Affiliations:** 1 Plasma Cell Dyscrasias Unit, Department of Clinical Therapeutics, National and Kapodistrian University of Athens, School of Medicine, Athens, Greece; 2 Applied Cancer Research and Drug Discovery, Translational Genomics Research Institute, City of Hope Cancer Center, Phoenix, AZ USA; 3 Department of Hemato-Oncology, University Hospital Ostrava, and Faculty of Medicine, University of Ostrava, Ostrava, Czech Republic; 4 Tisch Cancer Institute, Icahn School of Medicine at Mount Sinai, New York, NY USA; 5 Department of Hematology, Cancer Center Amsterdam, Amsterdam University Medical Center, VU University Amsterdam, Amsterdam, The Netherlands; 6 Department of Lymphoma and Myeloma, MD Anderson Cancer Center, Houston, TX USA; 7 Department of Hematology, University Hospital of Salamanca, IBSAL, CIC, IBMCC (USAL-CSIC), Salamanca, Spain; 8 Myeloma Unit, Division of Hematology, University of Torino, Azienda Ospedaliero-Universitaria Città della Salute e della Scienza di Torino, Torino, Italy; 9 Department of Haematology, Oxford University Hospitals NHS Foundation Trust, RDM, Oxford University, NIHR BRC Blood Theme, Oxford, UK; 10 Department of Haematology, The Royal Marsden Hospital, and Division of Molecular Pathology, The Institute of Cancer Research (ICR), London, UK; 11 Leeds Cancer Centre, Leeds Teaching Hospitals Trust, Leeds, UK; 12 Department of Oncology, Hematology and Bone Marrow Transplantation with Section of Pneumology, University Medical Center Hamburg-Eppendorf, Hamburg, Germany; 13 Department of Medicine, Division of Blood and Marrow Transplantation, Moores Cancer Center, University of California San Diego, La Jolla, CA USA; 14 Millennium Pharmaceuticals, Inc., a wholly owned subsidiary of Takeda Pharmaceutical Company Limited, Cambridge, MA USA; 15 Department of Hematologic Oncology and Blood Disorders, Levine Cancer Institute, Charlotte, NC USA

**Keywords:** Myeloma, Quality of life, Adverse effects, Cancer therapy, Myeloma

## Abstract

Treatment options in multiple myeloma (MM) are increasing with the introduction of complex multi-novel-agent-based regimens investigated in randomized clinical trials. However, application in the real-world setting, including feasibility of and adherence to these regimens, may be limited due to varying patient-, treatment-, and disease-related factors. Furthermore, approximately 40% of real-world MM patients do not meet the criteria for phase 3 studies on which approvals are based, resulting in a lack of representative phase 3 data for these patients. Therefore, treatment decisions must be tailored based on additional considerations beyond clinical trial efficacy and safety, such as treatment feasibility (including frequency of clinic/hospital attendance), tolerability, effects on quality of life (QoL), and impact of comorbidities. There are multiple factors of importance to real-world MM patients, including disease symptoms, treatment burden and toxicities, ability to participate in daily activities, financial burden, access to treatment and treatment centers, and convenience of treatment. All of these factors are drivers of QoL and treatment satisfaction/compliance. Importantly, given the heterogeneity of MM, individual patients may have different perspectives regarding the most relevant considerations and goals of their treatment. Patient perspectives/goals may also change as they move through their treatment course. Thus, the ‘efficacy’ of treatment means different things to different patients, and treatment decision-making in the context of personalized medicine must be guided by an individual’s composite definition of what constitutes the best treatment choice. This review summarizes the various factors of importance and practical issues that must be considered when determining real-world treatment choices. It assesses the current instruments, methodologies, and recent initiatives for analyzing the MM patient experience. Finally, it suggests options for enhancing data collection on patients and treatments to provide a more holistic definition of the effectiveness of a regimen in the real-world setting.

## Introduction

Today’s physicians treating multiple myeloma (MM) are faced with the challenge of individualizing treatment choices associated with the highly diverse patient populations seen across all treatment settings. Historically, median overall survival (OS) for MM patients was only ~3 years^1^, and there were a limited number of agents/regimens available. Now there is an increasing range of highly active treatment options available, offering novel combinations and leading to the marked improvements in patient outcomes seen in randomized clinical trials over the past 15 years^2^. These changes in the MM treatment landscape make the current scenario much more complex, requiring physicians to weigh varying goals of treatment in different settings^3–5^. The objectives of this review are to provide a comprehensive summary of the key factors that determine treatment goals and drive treatment choices for patients—specifically, therapy-related factors impacting patient-reported outcomes (PROs) that are additional to those related to commonly administered agents and other supportive pharmacologic interventions—and to summarize existing and emerging methodologies for capturing these drivers of treatment choices.

## The gap between the clinical-trial and real-world settings

Phase 3 studies remain the ‘gold standard’ for obtaining regulatory approvals, based on their strong internal validity, prespecified and well-defined endpoints, and use of randomization, blinding and control arms. Favorable efficacy and benefit:risk balances have been demonstrated in clinical trials for multiple new standard-of-care regimens in recent years. However, these prospective studies have limitations in terms of external validity and generalizability. Frequently, these clinical-trial data are first reported after a median follow-up of 1–2 years, and thus the ability of patients to continue treatment beyond the initial period is unknown. The increasingly complex novel-agent-based regimens are typically associated with toxicity additional to that arising from standard backbone agents such as dexamethasone, and in the real-world setting the feasibility of and adherence to these regimens may be more difficult. The full benefit may not be derived if drugs and regimens are not: (i) tolerable enough for real-world patients and may thus impact their quality of life (QoL), including for specific patient populations such as elderly/frail patients; (ii) available to patients, e.g., due to limited mobility or travel issues/preference, or due to affordability; or (iii) in line with patients’ preferences. There is a need for efficacious options that meet these criteria, and physicians require a balance of all relevant information when making treatment decisions.

Additionally, phase 3 studies may include an unrepresentative patient population. Many real-world and registry studies have concluded that approximately 40% of MM patients in the real world do not meet the criteria for inclusion in phase 3 studies on which approvals are based (Table 1)^6–11^. Patients may be ineligible for a range of reasons, including poor performance status, inadequate organ function, and adverse medical history or comorbidities, meaning that they are underrepresented in phase 3 clinical trials. As documented by these studies, clinical trial ineligibility is often associated with significantly poorer outcomes compared to those reported in trial-eligible patients, including shorter progression-free survival (PFS) and OS (Table 1)^6–11^. This leads to a lack of representative phase 3 trial data for this high proportion of real-world patients.

**Table 1:** Rates of clinical trial eligibility reported from real-world and registry studies of patients with MM.

Reference	Study/Registry	*N*	Region	Criteria used	Ineligible patients, %	Outcomes: ineligible vs eligible patients
Shah et al.^6^	Connect MM^®^	1406	United States	Common exclusion criteria from 24 RCTs, including absence of measurable disease, inadequate renal/hepatic/hematologic function, ECOG performance status 3/4, and history of other malignancy	40.0–56.8^a^	3-Year OS: 63% vs 70% (HR 0.81, 95% CI 0.67–0.99, *p* = 0.0392)
Fiala et al.^8^	CoMMpass	848	76 Sites worldwide, primarily in the United States, Canada, Italy, and Spain^†^	ECOG performance status 3/4, inadequate renal/hematologic function	22	91% Increase in risk of progression or death in ineligible patients (adjusted HR 1.91, 95% CI 1.45–2.52, *p* < 0.0001)
Chari et al.^7^	EHR database analysis	1265	United States	Eligibility criteria for ASPIRE, TOURMALINE-MM1, POLLUX, ELOQUENT-2, CASTOR, ENDEAVOR	47.9–72.3	With Rd treatment: 3-year OS: 63.5% vs 74.7% (mortality HR 1.46, 95% CI 1.03–2.07, *p* = 0.0329) With Vd treatment: 3-year OS: 46.2% vs 61.0% (mortality HR 1.51, 95% CI 1.14–2.01, *p* = 0.0045)
Knauf et al.^9^	German Tumour Registry Lymphatic Neoplasms	285	Germany	Heart failure, renal failure, other renal diseases, liver diseases, polyneuropathy, HIV positive	32	3-Year PFS: 18.8% vs 39.9% 3-year OS: 44.4% vs 69.4% 2-year DSS: 69.3% vs 88.4%
Hungria et al.^10^	INSIGHT MM	3201	EMEA 50.4%, North America 31.3%, Latin America 11.5%, Asia-Pacific 6.8%	20 Standard RCT ineligibility criteria, including inadequate renal/hepatic/hematologic function, ECOG performance status >2, hypercalcemia, cardiac medical history, chronic pulmonary disease, cerebrovascular disease, and history of other malignancy	39.2	Median TTNT (patients with CRAB symptoms): NDMM patients: 18.0 months vs NE RRMM patients: 14.9 vs 21.2 months
Klausen et al.^11^	Danish Multiple Myeloma Registry	1425	Denmark	Common eligibility criteria for non-transplant/elderly patients in the HOVON 87, VISTA, FIRST, and SWOG S0777 phase 3 studies	63.4	Median OS: 21.3 vs 44.0 months (HR 1.7, 95% CI 1.5–1.9, *p* < 0.0001)
				Common eligibility criteria for non-transplant/elderly patients in the ALCYONE phase 3 study	66.2	HR 1.7, 95% CI 1.5–1.9, *p* < 0.0001
		764	Denmark	Common eligibility criteria for transplant-eligible patients in the STaMINA, IFM2013–04, IFM DFCI 2009, and EMN02 phase 3 studies	54.8	Median OS: 65.5 vs 78.4 months (HR 1.3, 95% CI 1.0–1.5, *p* = 0.021)

^a^Dependent on criteria applied to the analysis. ^†^Information at time of submission from https://themmrf.org/finding-a-cure/our-work/the-mmrf-commpass-study/.

*CI* confidence interval, *CRAB* hypercalcemia, renal impairment, anemia, bone disease; *DSS* disease-specific survival, *ECOG* Eastern Cooperative Oncology Group, *EHR* electronic health record, *EMEA* Europe, the Middle East, and Africa; *HR* hazard ratio, *NDMM* newly diagnosed multiple myeloma, *NE* not estimable, *OS* overall survival, *PFS* progression-free survival, *RCT* randomized controlled trial, *Rd* lenalidomide-dexamethasone, *RRMM* relapsed/refractory multiple myeloma, *TTNT* time to next therapy, *Vd* bortezomib-dexamethasone.

## Additional considerations in real-world patients

**Fig. 1:**
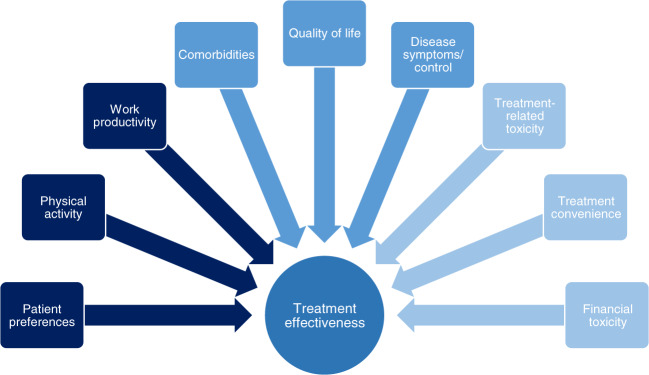
Treatment factors of importance to MM patients. There are multiple factors of importance to MM patients regarding their treatment that impact on the effectiveness of that treatment in the real-world setting.

In patients who are underrepresented in phase 3 clinical studies, treatment decisions must be based on additional considerations. We review multiple factors in addition to the traditional definition of efficacy that are key when considering the real-world MM patient experience (Fig. 1)^12–14^, including PROs. Those that may affect a patient’s health-related QoL in the real-world setting include their disease symptoms and how they are controlled, including supportive care, adverse events (AEs) associated with therapy, and pre-existing comorbidities. Also important to patients is their ability to participate in daily activities, the support available to them, access to treatment, and—particularly for elderly/frail patients—access to treatment centers^12–14^. The level of data captured on such patient-focused outcomes is another limitation of prospective phase 3 clinical studies.

We highlight that the relative importance of these different factors, and the goals of treatment, differ between patient groups and treatment settings—suggesting that ‘efficacy’ does not necessarily mean the same thing to different patients and depends on the balance of all attributes of a drug or regimen. In this context, a holistic needs assessment is valuable for making treatment choices, with broad support from a multidisciplinary team in the clinic and at home, and will assist in defining efficacy/effectiveness for each individual patient^15,16^. Additionally, in order to fully capture the patient’s experience of their MM treatment, it is necessary to be able to analyze and—where feasible—quantify all the relevant real-world drivers; we therefore also review the various instruments and studies developed to capture treatment impact/burden and preferences.

## Factors of importance to patients in the real-world setting

### Symptom burden

Among the hematologic malignancies, MM patients have the greatest symptom burden^17^. Symptoms related to the CRAB criteria (hypercalcemia, renal impairment, anemia, and bone disease) can be debilitating and may require supportive therapy such as bisphosphonates or denosumab^18^. These symptoms, along with fatigue, pain, gastrointestinal symptoms^16,19–21^, and other common disease-related complications such as neuropathic symptoms, as well as side effects that may arise from supportive therapy, can result in MM patients having significantly impaired QoL compared to the general population^16,19,20,22–24^. This highlights the need for rapid symptom control and minimal toxicity when choosing a treatment. However, in many clinical trials, patients with only biochemical progression are overrepresented.

### Side effects

Real-world studies of patients’ preferences have highlighted side effects of treatment as an important consideration. Various specific toxicities have been identified as being associated with specific agents, including peripheral neuropathy with bortezomib^25^ and thalidomide^26^, fatigue with lenalidomide^27^, cardiovascular side effects with carfilzomib^28,29^, gastrointestinal and hematologic side effects with ixazomib^30,31^, lenalidomide^26,32^ and panobinostat^33,34^, and fluid-retention effects, bone loss, eye complications and insomnia with corticosteroids^16^, with associated QoL decrements having been reported due to some of these toxicities. Real-world analyses have identified the substantial role played by toxicities in treatment discontinuation in both the frontline setting^35,36^ and more so in later lines^36^, suggesting that toxicities are more burdensome and limit the duration of treatment more substantially in the real-world setting compared with pivotal phase 3 studies. Such shortening of treatment duration due to toxicity has been shown to adversely impact outcomes^37^, highlighting how safety is an important component of efficacy.

### Daily activities

Multiple reports have demonstrated the value to patients of being able to continue with activities of daily living and of maintaining good physical and mental well-being. Impairment of activities of daily living due to MM and its treatment or other comorbidities is associated with poorer prognosis, as demonstrated by analyses of outcomes according to frailty indices^38,39^, as well as patient frustration^40^. The ability to continue with one’s daily routine and physical activities while receiving treatment is associated with fewer side effects and lower fatigue and is appreciated by patients as it improves QoL^41–43^.

These findings highlight the importance of gathering PROs in the context of considering the efficacy of a treatment regimen and taking into consideration the value patients place on being able to continue with their regular lives as much as possible. The associated mental health and well-being of the patient should be considered too^14,44^, as adverse impacts on a patient’s activities and emotional functioning may curtail treatment duration and effectiveness.

### Financial toxicity

Another aspect of concern to MM patients is the cost of treatment^12,14,21,44–47^, although the importance of this varies substantially worldwide according to healthcare system and access to drugs. Financial hardship may result from direct out-of-pocket costs arising from treatment and its side effects, depending on the healthcare system, and other indirect costs such as those involved in attending appointments (e.g., travel costs) and any compensation loss arising from impaired ability to work^46,47^. Studies have shown that such issues impact patients’ QoL^12,14,44^. Thus, treatment effectiveness may also be dependent on a patient’s ability to cope with the financial toxicity associated with receiving their regimen on a long-term basis.

### Treatment convenience/route of administration

Of related importance to patients is the convenience of treatment. While some patients may value the regular face-to-face contact with their treating physician/care team required with parenterally administered medications, some prefer oral medications even in the context of shorter progression-free time and/or more AEs^48^. This may be driven by various reasons; for example, patients may not be able to travel to infusion centers for treatment, due to limited mobility or distance from the clinic, they may wish to avoid the clinic/hospital setting due to specific circumstances, or they may want to minimize treatment burden associated with frequent hospital/clinic visits. Recent analyses have indicated patients’ preference for oral treatment is based on greater convenience, less impairment of daily activities, and less impact on work/productivity^48–51^. In this context, the feasibility of receiving treatment at home may be a relevant consideration, particularly in the current COVID-19 pandemic, with some studies showing domestic administration of therapy in MM patients spared the burden of repeat hospital transfers, leads to a low rate of treatment discontinuation^52^, and substantially improved QoL.

### Different patients, different perspectives: patient preferences in the real-world setting

Patients are becoming increasingly involved in their own treatment decision-making^53^, and their specific preferences, including the importance they attach to each of the factors discussed in the previous section, as well as their overall treatment goals, must be considered when selecting a regimen^54^. As MM is a heterogeneous disease with a heterogeneous patient population, these preferences and goals of treatment may differ between patients, depending on multiple patient-related, disease-related, and treatment-related factors^3–5,14,54,55^. ‘Efficacy’ therefore means different things to different patients. Treatment decision-making in the context of personalized medicine needs to be guided by an individual’s composite definition of what constitutes an effective treatment, per their preferences and treatment goals, in order to achieve the right balance between efficacy, safety, tolerability, feasibility, QoL, and treatment satisfaction^55^.

Within a specific patient population, drivers for treatment selection may be more granular in detail. For example, among younger MM patients, while some prioritize life expectancy/survival^44^, a discrete-choice experiment showed that others value preserving further treatment options, ‘not always thinking of the disease’, and treatment-free intervals as important characteristics of therapy, along with effectiveness^56^. Additionally, younger patients have been reported to rank severe or life-threatening toxicity as a greater concern than mild or moderate chronic toxicity more frequently than older patients, associated with the need to continue working and supporting their families^57^. However, younger, fitter patients may opt for an intensive treatment including stem cell transplantation in order to elicit a very deep response, improve their QoL, and achieve a lengthy remission and potential functional cure^58^.

In contrast, among elderly/frail MM patients, preferences may differ and factors of importance may be ranked differently. Frail patients may be older and/or have more comorbidities than fitter patients, and are at a greater risk of experiencing non-hematologic toxicity and of discontinuing treatment for reasons other than progression/death^38^. Furthermore, frail patients are less able to receive and tolerate intensive treatment approaches intended to induce deep responses^59^. Thus, for some of these patients, disease control and maintaining QoL may be priorities^2^, with comorbidities and the challenges of polypharmacy, potential toxicities associated with treatment, and functional limitations potentially weighted more heavily when making treatment decisions^60^. Treatment convenience and the ability to continue with daily activities may be of substantial importance in elderly/frail patients in the context of potentially receiving longer term, less-intensive treatment regimens than younger/fit patients.

As well as differing between groups of patients, preferences and weighting of factors of importance may also differ in the same patients at different stages of their treatment course. For example, among relapsed/refractory MM patients, a primary concern is the efficacy of their treatment regimen due to the desire to get their disease back under control after experiencing relapse. While QoL in newly diagnosed MM patients may be expected to increase during/following treatment, at relapse it may be expected only to stabilize^61^; therefore, QoL may perhaps be weighted less heavily when choosing treatment in these patients. Nevertheless, an underlying consideration for all treatment choices is that safety and tolerability are consistent drivers for efficacy, as the longer a patient can stay on treatment, the greater the therapeutic benefit they can accrue.

### Measurement of PROs: analyzing real-world preferences and factors of importance

In the context of implementing PROs, it is imperative to explore whether current QoL reporting and PRO methodologies for QoL data^62^ reflect all the key aspects of importance to patients and the impacts of novel treatments^63^. Over the past two decades a range of PRO measures (PROMs), including the European Organisation for the Research and Treatment of Cancer Quality of Life Questionnaire–Core-30 (EORTC QLQ-C30) and MM-specific (EORTC QLQ-MY20) instruments, among others (Table 2), have been developed and validated for MM, and a number of different types of studies have been evaluated as ways of capturing patients’ treatment preferences, experience, and perspectives (Table 3)^12–14,44,45,48,56,57,64–67^. Multiple studies have shown the beneficial impact of better QoL assessed using these PROMs on outcomes in MM, including OS (Table 3), highlighting that the use of PROMs in randomized clinical trials in MM provides valuable data. PROMs are particularly valuable in the context of randomization as this reduces the impact on PROs of differences in patient-/disease-related factors (although the potential for positive bias in open-label studies and due to data being gathered from those patients ‘doing best’ on the therapy should be acknowledged)^22,68–72^.

**Table 2:** Patient-reported outcome measures (PROMs), physician-completed instruments, and types of studies/patient-reported experience measures (PREMs) for capturing MM patients’ treatment preferences and factors of importance to their treatment experience.

Instrument/study type	Factors addressed	Scope/methodology
EORTC-QLQ-C30^68^	QoL, including physical and emotional functioning, symptoms, and toxicity Financial toxicity	30-Item instrument: Global Health Status/QoL scale 5 Functional scales (physical, role, cognitive, emotional, social) 3 Symptom scales (fatigue, nausea and vomiting, pain) 6 Single-item scales (appetite loss, diarrhea, dyspnea, constipation, insomnia, financial impact)
EORTC-QLQ-MY20^30^	QoL, including physical and emotional functioning, symptoms, and toxicity	20-Item instrument: 4 Domains (disease symptoms, side effects of treatment, body image, future perspectives)
FACT-MM^103^	Symptoms Physical activities Emotional well-being	14-Item instrument covering symptoms, impact on physical activities, and emotional well-being selected by expert clinicians and MM patients
MyPOS^19^	Symptoms Physical activities Emotional well-being Supportive care Financial toxicity	19-Question instrument covering 11 specific symptoms and questions regarding physical activities, emotional well-being, and supportive care
EQ-5D-3L/5L^19^ Time-trade-off utility measure	Physical activities Emotional well-being QoL	5-Item instrument (mobility, self-care, usual activities, pain/discomfort, anxiety/depression), plus visual analog scale for Global Health Status
Katz ADL scale^38^	Physical functioning / activities	6-Item instrument capturing whether a patient can/cannot perform key self-care activities
Lawton IADL scale^38^	Physical functioning / activities	8-Item instrument capturing whether a patient can/cannot perform key routine activities
Brief Pain Inventory	Pain and impact on physical functioning / activities and emotional functioning / QoL	9-Question (short form) or 32-question (long form) instrument evaluating current, overall, and worst pain experienced, and impact on activities, mood, and QoL
Stated preference study^57^	Patient preference	Multi-criteria decision analysis study; patients state preferences for a range of specific attributes of treatment
Direct preference assessment^56,64^	Weighting of factors of importance	Multi-attribute / multi-characteristic analysis study; patients rate importance of each attribute / characteristic
Discrete-choice experiment^48,56,64^	Patient preference	Multi-attribute / multi-characteristic analysis study; patients indicate treatment preferences between discrete sets of pairs of characteristics
Time-trade-off analysis^65^	Patient preferences	Multi-criteria valuation study; patients provide preferences for a range of health states for a specific time period or dying, and state the proportion of remaining time alive they would trade to be in full health, without a specific state
Value-based framework^66,67^	QoL	Evidence-based frameworks for decision-making, incorporating differing levels of patient experience data

*ADL* activities of daily living, *EORTC* European Organization for Research and Treatment of Cancer, *FACT-MM* Functional Assessment of Cancer–Multiple Myeloma, *IADL* instrumental activities of daily living, *MyPOS* myeloma patient outcome scale, *QLQ-C30/MY20* quality of life questionnaire Core 30 module / Myeloma 20-question module.

**Table 3:** The importance of PRO data in relation to outcomes in MM.

Study	Regimen	Setting	*N*	PRO data and impact on outcomes
Ludwig et al.^22^	ITd-I	RRMM	90	Significantly longer PFS (median 10.2 vs 6.6 months) and OS (median not reached vs 22.9 months) in patients with higher vs lower (dichotomized around median) Global Health Status/QoL score on EORTC-QLQ-C30 at baseline Significantly longer OS (median not reached vs 22.9 months) in patients with higher vs lower (dichotomized around median) physical functioning score at baseline
Strasser-Weippl et al.^71^	–	NDMM		Psychosocial QoL scores – role functioning, emotional functioning, social functioning, cognitive functioning – prognostic for OS
Viala et al.^72^	Bortezomib	RRMM	202	15 PRO parameters from the EORTC QLQ-C30, EORTC QLQ-MY24, FACIT-Fatigue, and FACT/GOG-NTx instruments were significant predictors for mortality on univariate analysis Fatigue (OR 1.052) and physical functioning (OR 0.964) from EORTC QLQ-C30 were significant predictors for mortality on multivariate analysis Predictive power for mortality of clinical variables was increased by addition of PRO variables
PROFILES registry^68^	–	MM	226	EORTC QLQ-C30 summary score associated (HR 0.89) with all-cause mortality in MM patients Global Health Status/QoL scale (HR 0.90) and physical functioning scale (HR 0.90) also associated with all-cause mortality in MM patients
SEER-MHOS analysis^70^	–	Elderly NDMM	521	Self-reported health using the SEER-MHOS instrument dichotomized as ‘high’ or ‘low’ Risk of all-cause (HR 1.32) and MM-specific (HR 1.22) mortality elevated in patients with ‘low’ vs ‘high’ self-reported health
NMSG 4/90 analysis^69^	MP	NDMM	524	Global Health Status/QoL, physical functioning, role functioning, cognitive functioning, fatigue, and pain domain scores on EORTC QLQ-C30 were statistically significant predictors of survival on univariate analysis. Poor physical functioning and cognitive functioning remained significant predictors of survival on multivariate analysis.

*EORTC QLQ-C30/MY20* European Organisation for the Research and Treatment of Cancer Quality of Life Questionnaire Core-30/Myeloma-specific module, *FACT-BMT* Functional Assessment of Cancer Therapy – Bone Marrow Transplant instrument, *HR* hazard ratio, *IADL* instrumental activities of daily living, *ITd-I* ixazomib-thalidomide-dexamethasone plus ixazomib maintenance, *KPS* Karnofsky performance status, *MM* multiple myeloma, *MP* melphalan-prednisone, *NDMM* newly diagnosed multiple myeloma, *NMSG* Nordic Myeloma Study Group, *OS* overall survival, *PFS* progression-free survival, *PRO* patient-reported outcome, *QoL* quality of life, *RRMM* relapsed/refractory multiple myeloma, *SEER-MHOS* Surveillance, Epidemiology, and End Results – Medicare Health Outcomes Survey; *TTP* time to progression.

It is possible that some aspects of QoL of importance to MM patients are not being captured with the current instruments, e.g., sexual functioning^73^. Further, QoL data obtained using these instruments often show limited differences between treatment arms, despite significant efficacy and safety profile differences, or do not reflect specific troublesome aspects of the safety profile of a regimen, effects also reported in other malignancies^26,61,74^. Therefore, it should be questioned whether these and other currently available tools have sufficient sensitivity to the aspects that are of most importance to MM patients’ QoL^63^. This may be particularly relevant if a novel agent or regimen is specifically associated with a toxicity infrequently reported with other agents, e.g., peripheral neuropathy with bortezomib or thalidomide^25,26^. In such instances, discrepancies may be seen between broader and more toxicity-specific PRO tools as well as between physician-reported and patient-reported side effects^25,75^.

These observations have led to an increasing focus on how to implement PROs routinely and accurately in clinical practice^76^, and on developing a set of standardized outcome measures. Use of a standard set of PROMs would enable broader consideration of the specific factors/drivers associated with treatment effectiveness and the risk:benefit ratio for a specific therapy, thereby improving treatment decision-making for individual patients.

A number of initiatives are underway worldwide that aim to quantify the additional factors associated with treatment satisfaction in MM^73,77^. One such initiative, the IMPORTA project, has developed a suggested core set of outcome measures and instruments for routine collection in patients with newly diagnosed MM^73^, including conventional clinical measures of efficacy and safety plus multiple PROs covering QoL, preferences, and treatment satisfaction with a recommended collection schedule for these outcomes^73^.

In addition to accurately capturing all factors associated with treatment satisfaction, frequency of PRO collection is of importance with regards to capturing data in a timely manner and maximizing sensitivity to changes in QoL and patient satisfaction—if the recall window is too lengthy, treatment impacts over the time period may not be fully captured. Administration of each PRO instrument needs to be sufficiently frequent to fully capture patients’ experience. PRO deterioration has been shown to precede clinical disease progression^22,23^, and so prospective observational studies using multiple QoL, preference, and satisfaction instruments to gather novel information may aid in routine patient management^77,78^.

### Real-world effectiveness

The definition of efficacy is ‘the ability to produce a desired or intended result’. In the context of clinical investigations, efficacy is used to define performance under ideal, controlled conditions, and primary efficacy endpoints are typically reported upfront, with secondary endpoints of safety, tolerability, and PROs providing support for the utility of novel regimens. In contrast, effectiveness refers to the performance of a regimen under real-world conditions. Consideration of PROs and their potential impact on efficacy may be more critical in the real-world setting compared to the potentially more motivating environment of clinical trial participation. Data from randomized controlled trials may not always reflect results for patients undergoing treatment in routine clinical practice, although data on some regimens indicate that duration of therapy, PFS, and/or time to next therapy are maintained between real-world non-clinical-trial data and clinical-trial data^79–82^, suggesting their efficacy translates into broader effectiveness^83^. There are multiple potential reasons for this ‘efficacy–effectiveness gap’^80,83^, and real-world effectiveness is dependent on multiple other endpoints beyond what is measured in clinical studies^13^, all of which must be considered in order to produce the desired or intended result. This suggests that, in the absence of head-to-head comparisons in clinical trials, indirect comparisons of regimens through the analysis of a single clinical trial efficacy endpoint, as is often done via network meta-analysis, may not accurately extrapolate to comparative real-world effectiveness.

Furthermore, differences between clinical trial and real-world outcomes must be considered in the context of the heterogeneous healthcare systems that exist between different countries and sometimes within individual countries. Differences in patient management and MM specialization between treatment centers may affect treatment outcomes, associated with factors beyond conventional efficacy and safety as determined in a clinical trial^80,84^. In fact, data on real-world treatment within specialist networks^81^ or at specialist MM centers^79^ demonstrate prolonged outcomes versus, for example, claims data analyses from broader real-world practice^85^. One potential driver for the discrepancy in outcomes between centers participating in clinical trials and MM specialist centers, compared to broader real-world practice, may be the timing of treatment initiation at relapse. Several studies have shown that delaying treatment until symptomatic relapse occurs, compared with starting therapy at biochemical relapse, identified through regular follow-up and monitoring, may result in poorer outcomes^79,86,87^. Additionally, differences in clinicians’ experience with new regimens and in the availability of infrastructure for monitoring and toxicity management may also be relevant.

In the context of the above, routine collection of additional endpoints such as QoL and other PROs^88,89^, plus healthcare resource utilization, alongside the key efficacy and safety data collected in clinical trials, would aid in providing a complete picture of efficacy of a regimen^90–94^. Furthermore, as reviewed recently, there is a need to improve safety assessment and reporting in various hematologic malignancies^95^, with improved data collection and evaluation aiming to provide additional valuable information of relevance to the real-world effectiveness of treatments. An additional element to consider in this expanded framework of standard data collection on MM patients is the incorporation of real-world data to augment the findings of randomized controlled trials. This would further enhance the datasets available on different treatment options. More informed decisions could thus be made between treatments that include clinical trial efficacy and safety data, QoL data, patient preferences and treatment satisfaction data, economic information, and other important issues.

### Future perspective and recommendations

**Fig. 2:**
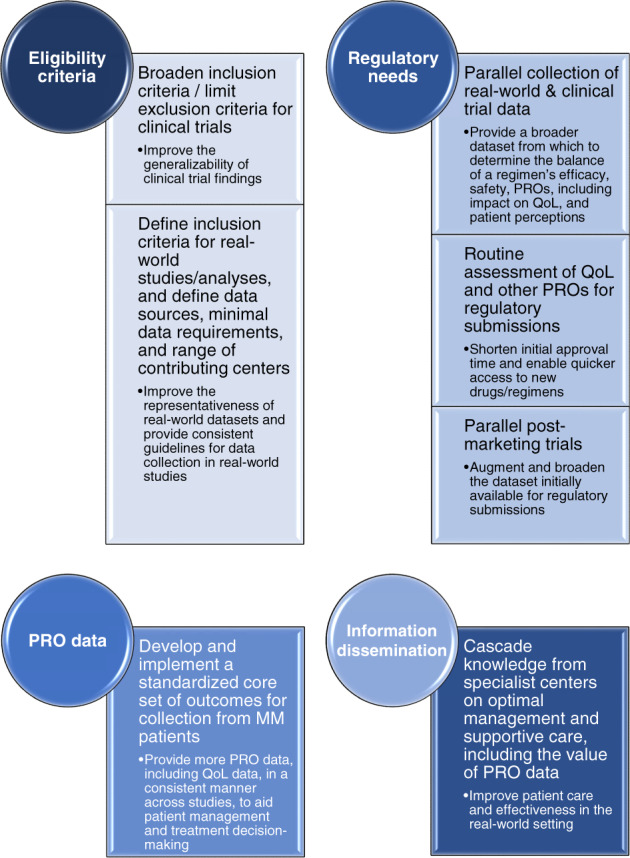
Enhancing MM data collection. Recommendations for enhancing future data collection on MM treatment regimens. MM, multiple myeloma; PRO, patient-reported outcome; QoL, quality of life.

With our increasing understanding of the differences between clinical trial patients and real-world populations, and the apparent gap between clinical trial efficacy and real-world effectiveness^80^, there are a number of recommendations that could enhance data collection on MM treatment regimens in the future (Fig. 2). For clinical trials there are ongoing initiatives aimed at modifying standard inclusion and exclusion criteria, for example, by removing comorbidity restrictions, which will improve the generalizability of trial findings^96,97^. Similar initiatives are also required for defining inclusion criteria for real-world evidence studies/analyses. Additional considerations include whether to obtain data from insurance databases or hospitals, determining the minimal requirements for clinical data, and evaluating whether to mimic the approach of cancer registries by incorporating data from smaller, non-specialist centers or offices and including all patients seen in the contributing clinic.

From the perspective of regulatory needs, the parallel collection of real-world data and clinical-trial data should be explored in order to provide a broader dataset on which to consider approval of a regimen. Incorporating routine assessment of QoL and other PROs within a broader context of a regimen’s efficacy may prove beneficial in making drugs available to patients more quickly, as many of these data are of relevance from a regulatory perspective^88,98,99^. Another approach that could help speed up patient access to new regimens would be to consider parallel post-marketing trials to gather additional data to augment the dataset initially available for regulatory submissions. However, in this context it will be important to explore the current challenges with real-world data and limitations of real-world studies, and identify the need for additional or improved datasets from this setting. For example, data from registries often do not include all the data required for Health Technology Assessments, for which extra evidence is then requested.

Across both clinical trials and real-world studies, there is a need to utilize more PROMs—implementation of a standardized core set of outcome measures for collection from MM patients will help in this regard^73^. Similarly, data from other ongoing studies will be of interest in order to determine how best to use such PRO data on QoL, patient preferences, and patient satisfaction in the context of patient management and treatment decision-making^77^. However, it will be important to consider the time pressures that healthcare professionals are under in their daily clinics. Completion of multiple instruments and/or analysis of multiple PROMs is likely to be too time-consuming for most, and so collection of a core set of PRO data will require a practical but comprehensive/informative tool that does not take too much time to complete and analyze.

In light of the heterogeneous healthcare systems that can exist within individual countries, there is also a need to disseminate information more widely regarding the optimal management and supportive care of MM patients, including the value of gathering PROs, along with critical information on how to utilize different regimens and agents. Cascading this knowledge from MM specialist centers to non-specialist practices in which fewer MM patients are routinely seen will be of value. Outcomes observed in clinical trials may be impacted by suboptimal management in the real-world setting^100^. Thus, addressing this need may help partially close the gap seen between clinical trial efficacy and real-world effectiveness.

## Conclusions

In conclusion, it is important to emphasize that selection of treatment options requires a review of the individual patients’ characteristics along with careful understanding of the difference between efficacy and effectiveness, and open, honest communication with patients to appropriately define their preferences. In the modern era of MM therapy, with multiple treatment options in the frontline and relapsed settings, the isolated use of conventional clinical trial efficacy as a metric for comparisons between agents/regimens is not optimal. While efficacy data from randomized clinical trials remain the gold standard for defining the relative benefit of a regimen, safety, QoL, and other PROs are important contributors to a regimen’s effectiveness in the real world. Regimens that are more tolerable and convenient for patients and have a positive impact on patients’ QoL may be more likely to be administered for longer, thus enabling their effectiveness. For example, development of subcutaneous or oral instead of intravenous formulations of agents^52,101,102^, potentially enabling home administration^52,101^, or development of novel therapeutics within a drug class that have lower risks of key toxicities, while preserving the efficacy, are valuable in this context.

A broader scope of additional endpoints and patient-related factors must be considered due to their critical impact on the effectiveness of a treatment in the real world. Standardizing the collection and reporting of these endpoints and factors^89^, together with validation of novel instruments or composite metrics incorporating these additional considerations, will enable a broader comparison between different treatment regimens that is more meaningful to patients in the real-world setting. Elucidating how the weighting of each of the factors contributing to a regimen’s effectiveness differs between groups of patients may lead to more patient-focused decision-making for tailored treatment approaches. An ultimate goal would be deriving a convenient, patient-friendly way to measure these aspects in an individual patient in a time-efficient way for physicians.

A final point to emphasize is that going forward it will be necessary to ensure that patients are more representative of the real-world MM patient population, both in clinical trials and in real-world studies/analyses. Such approaches will further support a comprehensive characterization of efficacy, safety, and PROs, including impact on QoL, associated with a treatment regimen in a representative population, thereby enabling improved treatment decision-making and personalization of therapy.
